# Simultaneous Expression of CD70 and POSTN in Cancer-Associated Fibroblasts Predicts Worse Survival of Colorectal Cancer Patients

**DOI:** 10.3390/ijms25052537

**Published:** 2024-02-22

**Authors:** Masayuki Komura, Chengbo Wang, Sunao Ito, Shunsuke Kato, Akane Ueki, Masahide Ebi, Naotaka Ogasawara, Toyonori Tsuzuki, Kenji Kasai, Kunio Kasugai, Shuji Takiguchi, Satoru Takahashi, Shingo Inaguma

**Affiliations:** 1 Department of Experimental Pathology and Tumor Biology, Nagoya City University Graduate School of Medical Sciences, Nagoya 467-8601, Japan; komura@med.nagoya-cu.ac.jp (M.K.); c221904@ed.nagoya-cu.ac.jp (C.W.); c211705@ed.nagoya-cu.ac.jp (A.U.); sattak@med.nagoya-cu.ac.jp (S.T.); 2 Department of Gastroenterological Surgery, Nagoya City University Graduate School of Medical Sciences, Nagoya 467-8601, Japan; sunaoti.21@gmail.com (S.I.); takiguch@med.nagoya-cu.ac.jp (S.T.); 3 Division of Gastroenterology, Department of Internal Medicine, Aichi Medical University School of Medicine, Nagakute 480-1195, Japan; katou.shunsuke.668@mail.aichi-med-u.ac.jp (S.K.); ebi.masahide.814@mail.aichi-med-u.ac.jp (M.E.); ogasawara.naotaka.667@mail.aichi-med-u.ac.jp (N.O.); kasugai.kunio.527@mail.aichi-med-u.ac.jp (K.K.); 4 Surgical Pathology, Aichi Medical University School of Medicine, Nagakute 480-1195, Japan; tsuzuki@aichi-med-u.ac.jp; 5 Department of Pathology, Aichi Medical University School of Medicine, Nagakute 480-1195, Japan; kkasai@aichi-med-u.ac.jp; 6 Department of Pathology, Nagoya City University East Medical Center, Nagoya 464-8547, Japan

**Keywords:** colorectal cancer (CRC), immunohistochemistry, CD70, periostin (POSTN), cancer-associated fibroblast (CAF)

## Abstract

Colorectal cancer (CRC) is one of the most common gastrointestinal cancers worldwide, with high morbidity and mortality rates. The evidence for the tumor-supporting capacities of cancer-associated fibroblasts (CAFs) that modulate cancer cell proliferation, invasion, metastasis, and tumor immunity, including in CRC, has been attracting attention. The present study examined the expression status of CD70 and POSTN in CRC and analyzed their association with clinicopathological features and clinical outcomes. In the present study, in total 15% (40/269) and 44% (119/269) of cases exhibited CD70 and POSTN expression on CAFs, respectively. Co-expression of CD70 and POSTN was detected in 8% (21/269) of patients. Fluorescent immunohistochemistry identified the co-expression of CD70 and POSTN with FAP and PDPN, respectively. ACTA2 was not co-expressed with CD70 or POSTN in CRC CAFs. CRC with CD70+/POSTN+ status in CAFs was significantly associated with distant organ metastasis (*p* = 0.0020) or incomplete resection status (*p* = 0.0011). CD70+/POSTN+ status tended to associate with advanced pT stage (*p* = 0.032) or peritoneal metastasis (*p* = 0.0059). Multivariate Cox hazards regression analysis identified CD70+/POSTN+ status in CAFs [hazard ratio (HR) = 3.78] as a potential independent risk factor. In vitro experiments revealed the activated phenotypes of colonic fibroblasts induced by CD70 and POSTN, while migration and invasion assays identified enhanced migration and invasion of CRC cells co-cultured with CD70- and POSTN-expressing colonic fibroblasts. On the basis of our observations, CD70 and POSTN immunohistochemistry can be used in the prognostication of CRC patients. CRC CAFs may be a promising target in the treatment of CRC patients.

## 1. Introduction

Colorectal cancer (CRC) is one of the most common gastrointestinal cancers worldwide, with high morbidity and mortality rates [1]. The evidence for the tumor-supporting capacities of cancer-associated fibroblasts (CAFs) modulating cancer cell proliferation, invasion, metastasis, and tumor immunity in cancers including CRC has been attracting attention [2,3]. On the basis of their broad range of tumor-supporting abilities, CAFs are believed to be a promising target in cancer therapy.

CD70 is a type II transmembrane surface protein comprising 193 amino acids. CD70 is classified as a member of the tumor necrosis factor superfamily (TNFSF) based on its C-terminal tumor necrosis factor (TNF) homology domain [4,5]. CD70 expression is tightly regulated on activated T cells, B cells, and mature dendritic cells [6,7]. CD27, a physiological receptor for CD70, is a co-stimulatory immune checkpoint receptor that is constitutively expressed on a broad range of immune cells such as T cells, natural killer cells, and B cells. Under physiological conditions, the CD70–CD27 signaling pathway plays a co-stimulatory role in promoting T-cell expansion and differentiation through activation of the nuclear factor-κB pathway [7,8,9]. Aberrant CD70 expression has been reported to accelerate immune evasion and increase malignant phenotypes in many tumor types, including pleural mesothelioma [9,10,11,12,13,14,15].

Periostin (POSTN), a secreted extracellular matrix (ECM) protein, was originally identified in mesenchymal lineage cells such as osteoblasts. POSTN has been reported to play a critical role in development and tissue regeneration [16]. As well as bone development and remodeling [17], recent studies indicated indispensable roles for POSTN in the activation, differentiation, and contraction of fibroblasts in dermal regeneration and wound healing [18]. Aberrant POSTN expression with poor clinical outcome has been reported in solid epithelial malignancies including CRC [19,20,21,22,23,24]. During tumor progression, POSTN has been reported to play a critical role in the formation and remodeling of cancer tissue microenvironments via CAFs and accelerates cell adhesion, survival, invasion, angiogenesis, metastasis, and epithelial–mesenchymal transition (EMT) through interactions with tumor cell–surface receptor integrins such as αvβ3, αvβ5, and α6β4 to modulate intracellular signaling [19,21,25].

CAFs, a major component of the cancer stroma, are composed of a highly heterogeneous population of cells with different functions [26]. This heterogeneity probably originates from their different origins: bone-marrow-derived mesenchymal stromal cells [27], mature adipocytes [28], resident fibroblasts [29,30], and other cells exist within tumor microenvironments [31]. Tumor cells after the process of EMT have been suggested as another source of CAFs [31]. On the basis of the heterogeneity of CAFs, specific and common markers have not been identified; however, many attempts have been made to identify markers that can classify CAFs [32]. In CRC, according to gene expression status deduced from single-cell sequencing, CAFs have been classified into two types: CAF-A and CAF-B [33]. CAF-As have been specified by their expression of *FAP*, *MMP2*, *DCN*, and *COL1A2*, indicating their ECM-remodeling capacity. In contrast, CAF-Bs have been reported to express cytoskeletal genes with activated myofibroblast marker expressions [33].

Our group recently reported the survival implications of stromal CD70 [34] and POSTN [23] in CRC; however, the significance of the co-expression of CD70 and POSTN in CRC has not been analyzed, to our knowledge. The present study examined the expression status of both CD70 and POSTN in CRC and analyzed their association with clinicopathological features and clinical outcomes. Furthermore, the associations between CD70 and POSTN expression in CAFs and expression of immune cell markers and activated fibroblast markers were analyzed. In addition, molecular experiments were performed to elucidate the functions of CD70 and/or POSTN in human colonic fibroblasts.

## 2. Results

### 2.1. Expression of CD70 and POSTN in Non-Neoplastic Colonic Mucosa and CRC

**Figure 1:**
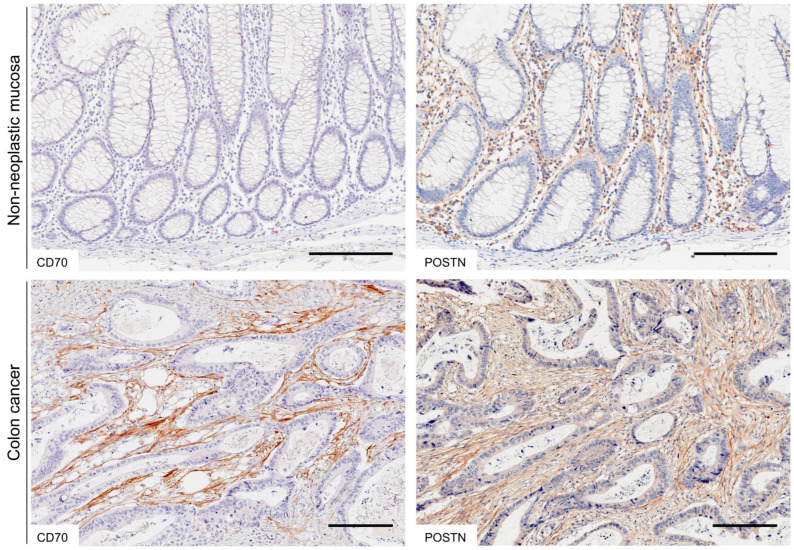
Representative images of CD70 and POSTN immunostaining in CRC. In non-neoplastic colonic mucosa, CD70 was weakly expressed on the immune cells. POSTN was expressed on the stromal cells. Both CD70 and POSTN were expressed on CRC CAFs. Bar, 200 μm.

In non-neoplastic colonic mucosae, both CD70 and POSTN were expressed at undetectable levels in epithelial cells. In contrast, CD70 and POSTN were weakly expressed in the immune and stromal cells within the lamina propria, respectively (Figure 1). In CRC cells, CD70 expression was detected in only 2.2% of cases (6/269). CRC cells expressed POSTN at undetectable levels.

**Figure 2:**
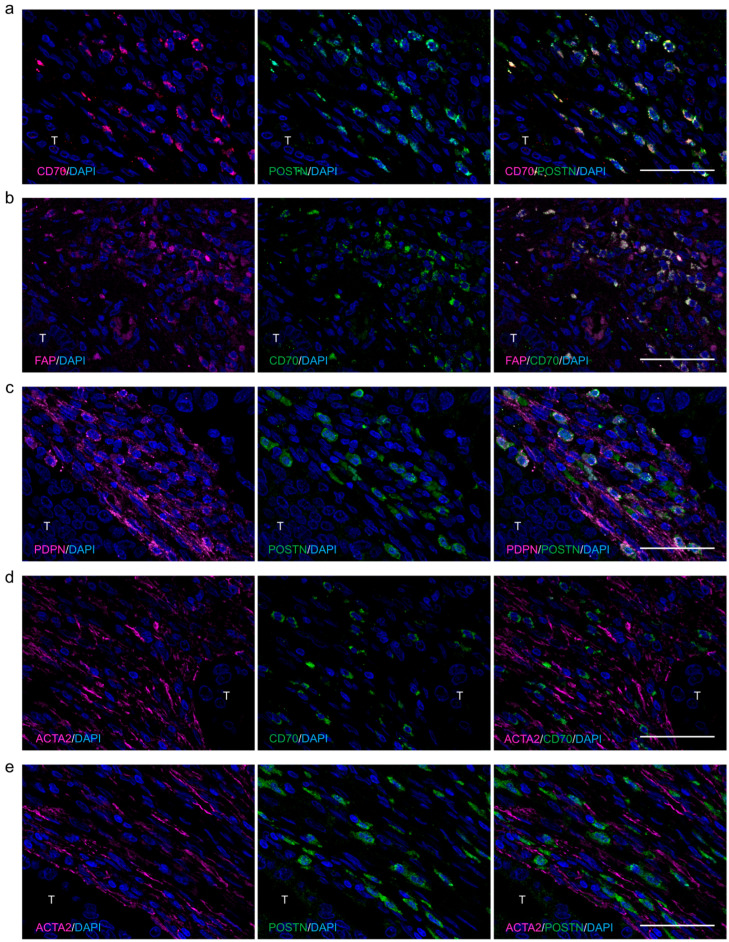
Representative images of fluorescent immunostaining in CRC. (**a**) Fluorescent immunohistochemistry identified co-expression of CD70 and POSTN on CRC CAFs. (**b**) CD70 and FAP were co-expressed in some of the CRC CAFs. (**c**) POSTN was co-expressed with PDPN in some. (**d**,**e**) ACTA2 was not co-expressed with CD70 (**d**) or POSTN (**e**) in CRC CAFs. T, tumor. Bar, 50 μm.

In CRC tissues, in total 15% (40/269) and 44% (119/269) of cases exhibited CD70 and POSTN expression on CAFs, respectively (Figure 1 and Figure 2, and Table 1). CD70 and POSTN co-expression in CRC CAFs was detected in 8% (21/269) of cases (Figure 2a and Table 1). Fluorescent immunohistochemistry identified the co-expression of CD70 and FAP in some of the CRC CAFs (Figure 2b). POSTN was co-expressed with PDPN (Figure 2c); however, ACTA2 was not co-expressed with CD70 or POSTN in CRC CAFs (Figure 2d,e).

### 2.2. Characteristics of CRC Classified According to CAF CD70 and POSTN Expression

The clinical, pathological, and immunohistochemical features of the analyzed tumors are summarized in Table 1 according to CD70 and POSTN expression. CRC with CD70+/POSTN+ status in CAFs was significantly associated with incomplete resection status (*p* = 0.0011) or distant organ metastasis (*p* = 0.0020). CD70+/POSTN+ status tended to be associated with advanced pT stage (*p* = 0.032) or peritoneal metastasis (*p* = 0.0059; Table 1).

### 2.3. Association of CAF CD70 and POSTN Expression with Cellular Proliferation Marker Expression, p53 Immunoreactivity, and KRAS/BRAF Mutations in CRC Cells

**Figure 3:**
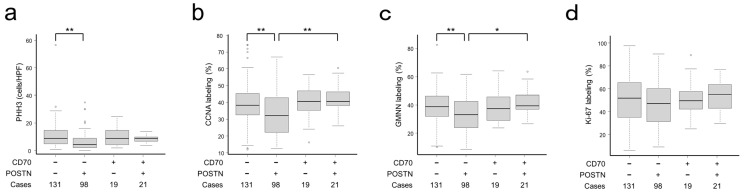
Cellular proliferation marker expression classified according to CD70 and POSTN expression. (**a**–**d**) CD70−/POSTN+ CRC showed significantly lower expression of PHH3 (**a**), CCDN (**b**), and GMNN (**c**). No significant association was detected between CD70 and POSTN expression status and Ki-67 (**d**). *, *p* < 0.05; **, *p* < 0.01. The circles indicate outliers.

To uncover the associations between CAF characteristics and the proliferative activities in CRC cells, cellular proliferation marker expressions in CRC cells were analyzed, following our previous study [35]. Representative images for cellular proliferation marker expressions are presented in Supplementary Figure S1a. In the association of cellular proliferation markers, CD70−/POSTN+ showed a significantly inverse association with phospho-histone H3 (PHH3), cyclin A (CCNA), and geminin (GMNN) expression but no significant association with Ki-67 labeling index (Figure 3).

No association was detected between CD70 and POSTN expression and tumor mutation status or p53 immune reactivity (Supplementary Table S1).

### 2.4. Association of CAF CD70 and POSTN Expression with Immune Cell and Stromal Marker Expression

**Figure 4:**
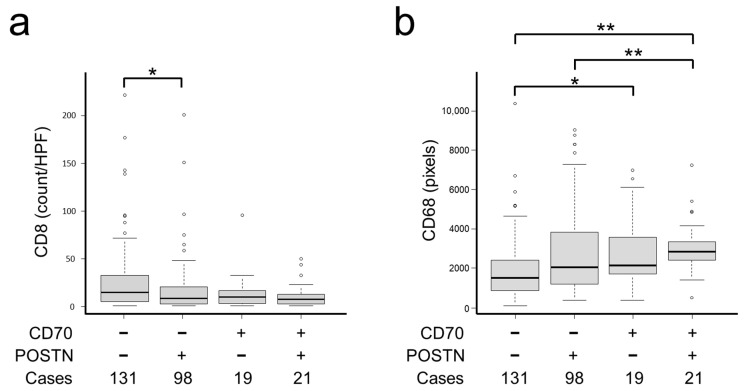
CAF CD70 and POSTN expression was significantly associated with immune cell infiltration. CD70 and POSTN were significantly associated with CD8- (**a**) and CD68-positive immune cells (**b**) in CRC. *, *p* < 0.05; **, *p* < 0.01. The circles indicate outliers.

Representative images of immune cells are presented in Supplementary Figure S1b. Regarding immune cells within the CRC microenvironment, CD70−/POSTN− CRC contained a significantly higher number of CD8+ immune cells (*p* = 0.0063; Table 2 and Figure 4a). In contrast, CD70+/POSTN+ CRC contained a significantly higher number of CD68+ immune cells (*p* < 0.0001; Table 2 and Figure 4b).

### 2.5. Survival Analyses of Patients with CRC

**Figure 5:**
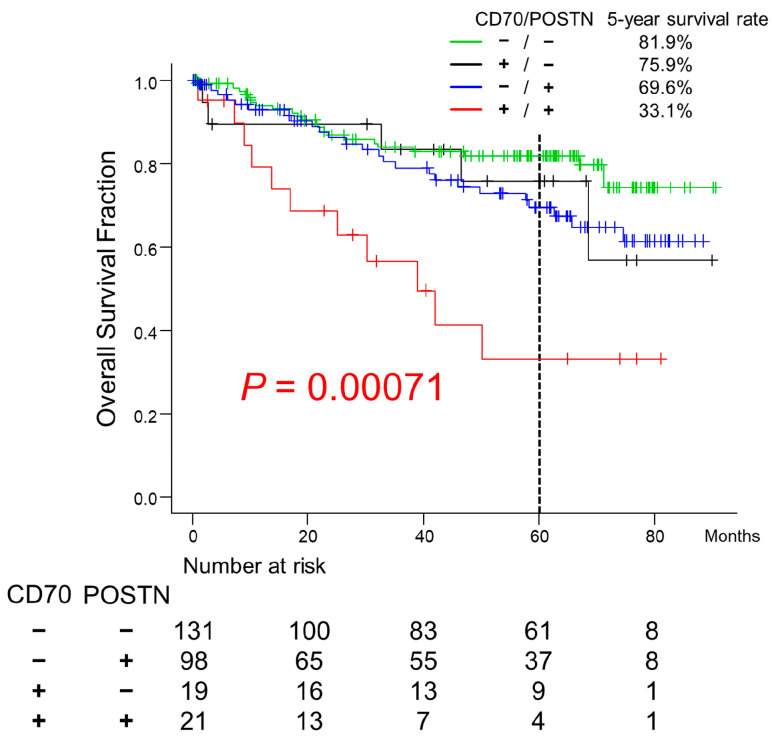
Overall survival of CRC patients classified according to CD70 and POSTN expression on CAFs. Patients with CRC exhibiting stromal CD70 and POSTN expression showed the worst clinical outcomes. The post hoc test identified significant differences as follows: CD70+/POSTN+ vs. CD70−/POSTN− (*p* = 0.00036); CD70+/POSTN+ vs. CD70−/POSTN+ (*p* = 0.028). Survival at 5-year was indicated by dashed line.

CRC patients with CD70+/POSTN+ CAFs had a significantly worse 5-year survival rate than other groups (33.1%; *p* = 0.00071; Figure 5), probably due to the frequent peritoneal and distant organ metastasis with incomplete resection status (Table 1). Multivariate Cox hazards regression analysis identified tubular-forming histology (hazard ratio [HR] = 0.25, 95% confidence interval [CI] = 0.13–0.47, *p* < 0.0001) and younger age (<70 years old, HR = 0.50, 95% CI = 0.29–0.86, *p* = 0.012) as potentially favorable factors. The analysis also revealed lymph node metastasis (HR = 1.98, 95% CI = 1.14–3.45, *p* = 0.015), co-expression of CD70 and POSTN in CAFs (HR = 3.78, 95% CI = 1.83–7.83, *p* = 0.00035), and peritoneal metastasis (HR = 5.45; 95% CI = 3.05–9.73, *p* < 0.0001) as potential independent risk factors for patients with CRC (Table 3).

### 2.6. CD70 and POSTN Induced Activated Phenotypes in Colonic Fibroblasts

**Figure 6:**
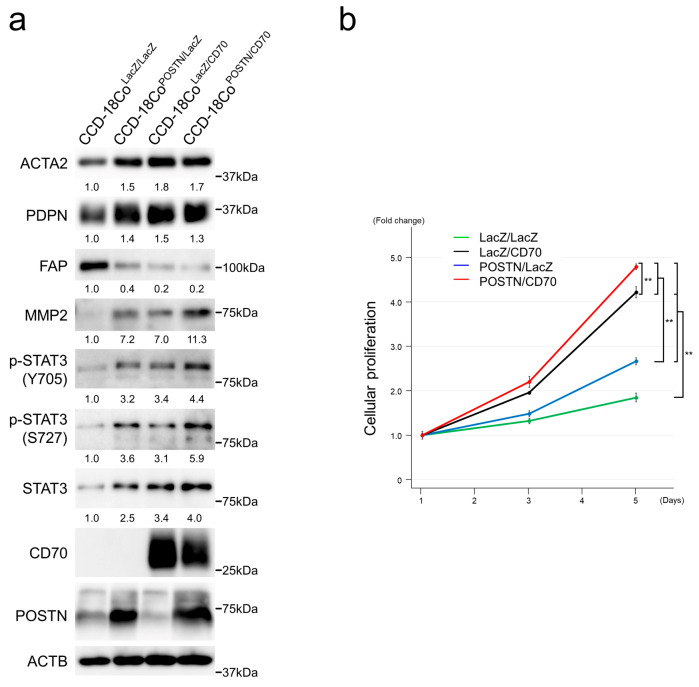
Effects of CD70 and POSTN on colonic fibroblasts. (**a**) Exogenous expression of CD70 and/or POSTN in human colonic fibroblast CCD-18Co upregulated ACTA2, PDPN, MMP2, and STAT3 expression, whereas FAP was downregulated. Additive effects of CD70 and POSTN were observed for MMP2, STAT3, and phosphorylated STAT3. Note that POSTN secreted into the culture medium was analyzed by immune blots. (**b**) Cellular proliferation of CCD-18Co was significantly upregulated by CD70 and POSTN. Assays were performed in triplicate. Data are presented as the mean ± SD. **, *p* < 0.01.

In the present study, CCD-18Co human colonic fibroblasts were lentivirally transduced for CD70 and POSTN expressions based on the notion that FAP-positive CAFs mainly originate from normal fibroblasts [36]. In CCD-18Co, both CD70 and POSTN upregulated ACTA2, PDPN, and MMP2. Furthermore, both CD70 and POSTN upregulated STAT3 expression with phosphorylation at tyrosine 705 (Y705) and serine 727 (S727) residues. Additive effects of CD70 and POSTN were observed in MMP2 and phosphorylated STAT3 under our experimental conditions. In contrast, FAP was downregulated in CD70- and/or POSTN-transfectants (Figure 6a).

To assess the functions of CD70 and POSTN in CCD-18Co cells, we performed cellular proliferation assays. CCD-18Co^LacZ/CD70^, CCD-18Co^POSTN/LacZ^, and CCD-18Co^POSTN/CD70^ exhibited enhanced cellular proliferation compared with control CCD-18Co^LacZ/LacZ^. CCD-18Co^POSTN/CD70^ showed the highest cellular proliferation among the transfectants analyzed (Figure 6b), with significance.

### 2.7. Colonic Fibroblasts Expressing CD70 and POSTN Enhanced the Migration and Invasion of Co-Cultured CRC Cells

Aberrantly activated stromal cells have been reported to accelerate the malignant phenotypes of cancer cells, including migration and invasion, by communicating with cancer cells [31,37,38,39]. In the present study, we attempted to elucidate the effects of colonic fibroblasts with CD70 and/or POSTN expression on the migration and invasion of co-cultured CRC cells.

**Figure 7:**
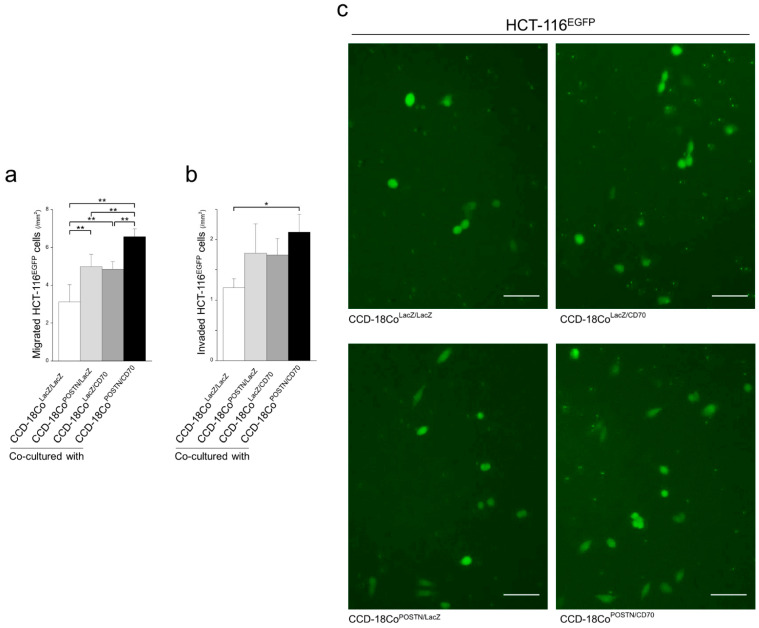
Co-culture migration and invasion assays. (**a**) The migration of EGFP-tagged HCT-116 cells was significantly enhanced when they were co-cultured with CCD-18Co^LacZ/CD70^, CCD-18Co^POSTN/LacZ^, and CCD-18Co^POSTN/CD70^. Assays were performed in triplicate. Data are presented as the mean ± SD. **, *p* < 0.01. (**b**) EGFP-labeled HCT-116 cells co-cultured with CCD-18Co^POSTN/CD70^ uniquely showed significantly more enhanced invasiveness than CCD-18Co^LacZ/LacZ^. Assays were performed in triplicate. Data are presented as the mean ± SD. *, *p* < 0.05. (**c**) Representative images of the results for migration assays. Bar, 200 µm.

In migration assays, the migration of HCT-116 cells was significantly enhanced in co-culture with CCD-18Co^LacZ/CD70^ and CCD-18Co^POSTN/LacZ^ compared with those co-cultured with control CCD-18Co^LacZ/LacZ^. In addition, additive effects of CD70 and POSTN were demonstrated (Figure 7a,c). In invasion assays, HCT-116 cells co-cultured with CCD-18Co^POSTN/CD70^ uniquely showed significantly enhanced invasiveness compared with CCD-18Co^LacZ/LacZ^ (Figure 7b).

## 3. Discussion

Classifications of the colonic fibroblasts and CRC CAFs using single-cell sequencing have been developing [26,33]. CRC CAFs are classified into two types according to their gene expression status: CAF-A expresses *DCN*, *FAP*, *MMP2*, and *COL1A2*, indicating its ECM-remodeling capacity; and CAF-B expresses cytoskeletal genes and markers of activated myofibroblasts such as ACTA2 [33]. We speculated that CD70+/POSTN+ CAFs corresponded to CAF-A on the basis of their co-expression with FAP but not with ACTA2. In line with our cohort study, in vitro experiments identified enhanced malignant potential of CRC cells co-cultured with CD70- and POSTN-positive colonic fibroblasts. Contrary to our expectations, exogenous expression of CD70 and POSTN on human colonic fibroblasts resulted in the enhancement of the intermingled characteristics of both CAF-A and CAF-B. These results may indicate the difference in originating cells for CAFs and/or the differential effects of CD70 and POSTN on these cells.

CAFs have been reported to regulate cancer cell invasion and metastasis through multiple mechanisms: (i) secretion of growth factors supporting cancer cells; (ii) remodeling of the ECM; and (iii) modulation of EMT in cancer cells [31,37,38,39]. In the present study, CD70+/POSTN+ status in CAFs was significantly associated with both incomplete resection status and distant organ metastasis in CRC patients. It also tended to be associated with peritoneal metastasis. We hypothesized that CAFs expressing CD70 and POSTN promoted the invasion and intravasation of the CRC cells at the primary site. In our in vitro experimental studies, both CD70 and POSTN induced enhanced cellular proliferation with activation of the STAT3 pathway, which is critical for fibroblast activation and tissue fibrosis [40,41,42,43]. Furthermore, MMP2 upregulation in CCD-18Co^POSTN/CD70^ indicated the upregulated matrix-remodeling capacity of the colonic fibroblasts, and co-culture migration and invasion assays revealed that CCD-18Co^POSTN/CD70^ enhanced the migration and invasion of co-cultured CRC cells. These observations may be due to secreted growth factors and/or chemoattractants from CCD-18Co expressing CD70 and POSTN. From the results of the co-culture invasion assays, we speculate that the ECM-remodeling capacity of CCD-18Co^POSTN/CD70^ may be involved. Targeting of CRC CAFs expressing CD70 and POSTN may be a promising therapeutic strategy for CRC patients.

CAFs are considered to induce an immunosuppressive tumor microenvironment through secreting cytokines, chemokines, and pro-angiogenic factors. In the present study, CD70−/POSTN− tumors contained significantly higher numbers of CD8+ immune cells, which have been considered as favorable factors [44]. In contrast, CRC CD70+/POSTN+ CAFs contained significantly higher numbers of CD68+ immune cells, indicating M1-polarized macrophage-dominant infiltration into the CRC microenvironment. M1 macrophages are characterized by their proinflammatory properties (such as secretion of IL-12 and reactive oxygen species) that promote antitumor T_H_1-type responses; therefore, infiltration of this type of immune cell was considered as a predictor of favorable clinical outcomes [44]. Based on our observations, we considered that the mechanism responsible for the worst clinical outcome in patients with CD70+/POSTN+ tumors cannot fully be explained by the immune cell contents. CD70- and/or POSTN-positive CAFs may have limited capacity for the modulation of the tumor immune microenvironment.

In the present study, CD70−/POSTN+ CAFs uniquely showed significantly lower proliferation rates in CRC. It was reported that CRC cell proliferation decreases according to pT stage [35]. In the present study, even in the stage-matched analyses, this type of tumor showed significantly lower proliferation rates. In an in vitro study, POSTN was reported to enhance the proliferation of CRC cells [23]. We consider that factor(s) other than POSTN in stromal cells dominantly regulate the proliferation of CRC cells in vivo.

The limitations of this study include the small number of CRC patients. A larger cohort with gene mutation and comorbidity information may be needed to identify the additional clinical significance of CD70 and POSTN expression in CRC. In the present study, three-color fluorescent immunohistochemical staining was performed in a representative case. Application of multi-color immunohistochemical staining to cohort analyses might be useful for the further characterization of CAFs. Another limitation was the unavailability of cultured CAFs. In the present study, fibroblasts of human non-neoplastic colonic origin were used as a model. The use of CAFs of human CRC origin may reveal additional findings.

The present study immunohistochemically evaluated the expression of CD70 and POSTN in CRCs. To best of our knowledge, the present study is the first to identify that CD70 and POSTN double-positive status in CRC CAFs is a potential risk factor for CRC patients. Furthermore, CD70 and POSTN induced activated phenotypes in colonic fibroblasts and accelerated the migration and invasion of co-cultured CRC cells in vitro. According to our observations, CD70 and POSTN immunohistochemistry can be used in the prognostication of CRC patients. CRC CAFs expressing CD70 and POSTN may be a promising target in the treatment of CRC patients.

## 4. Materials and Methods

### 4.1. Tissue Samples

The institutional ethical review boards of Aichi Medical University Hospital and Nagoya City University Graduate School of Medical Sciences approved this project without the need for patient consent by giving them the opportunity to opt out. Depending on the availability of formalin-fixed, paraffin-embedded (FFPE) samples and clinical information, two hundred sixty-nine primary colorectal adenocarcinomas resected at Aichi Medical University Hospital from 2009 to 2012 were selected. All of the patients were naïve to chemotherapy or radiotherapy. Patients were followed for up to 90 months after operation. All tumors were diagnosed as invasive according to TNM classification [45]. Tumors with glandular formation (>50%) or mucus production (>50% of the area) were defined as having a differentiated or mucus-producing histology. A single 4.5-mm core tumor tissue sample derived from an invasive area of the FFPE specimen was assembled into multitumor blocks containing up to 30 samples. Approximately 20% of cores contained an invasive front.

### 4.2. Immunohistochemistry

Immunohistochemistry was performed using a Leica Bond-Max (Leica Biosystems, Wetzlar, Germany) or Ventana BenchMark XT automated immunostainer (Roche Diagnostics, Basel, Switzerland). The antibodies used in the present study are summarized in Supplementary Table S2. Signals were visualized using 3,3′-diaminobenzidine. Collagens were stained with Picro-Sirius red stain (ScyTek Laboratories, Inc., Logan, UT, USA).

Immunohistochemistry data for CD70, POSTN, cellular proliferation, and immune cell markers were included in our previous study [23,34,35]. In brief, CD70-, POSTN-, CD68-, and CD163-positive areas were evaluated using ImageJ software 1.51k (NIH, Bethesda, MD, USA) [23,34]. The cut-off values were set according to our previous reports: CD70, 1000 pixels; and POSTN, 8328 pixels. The numbers of PHH3-, CD27-, CD4-, CD8-, PD-1, and FOXP3-positive cells were counted in a hot-spot area at a high-power field (×400). Ki-67, CCNA, and GMNN labeling indices were determined by counting more than 500 tumor cells per case in a high-power field (×400).

p53 immunoreactivity was classified as follows: wild-type, overexpression, complete loss, and cytoplasmic expression [46]. In the evaluation of complete loss of p53 expression, cases without internal controls such as fibroblasts and lymphoid cells were eliminated from the study.

Cases expressing all four mismatch repair (MMR)-related proteins (MLH1, PMS2, MSH2, and MSH6) in all the tumor cells were considered to be preserved MMR CRC.

### 4.3. Fluorescent Immunohistochemistry

Fluorescent immunohistochemistry was performed using a Leica Bond-Max (Leica Biosys-tems, Wetzlar, Germany). Signals were visualized using secondary antibodies labelled with fluorescein or tetramethylrhodamine applied at a dilution of 1:500 (Molecular Probes, Thermo Fisher Scientific K. K., Tokyo, Japan). Fluorescent images were taken using a FV3000 laser confocal scanning microscope (OLYMPUS, Tokyo, Japan).

### 4.4. Statistical Analyses

Statistical analyses were performed using EZR software version 1.41 [47]. The Chi-squared test, Fisher’s exact test, Cochran–Armitage trend test, Mann–Whitney U test, and Kruskal–Wallis test were performed to analyze the statistical correlations between categorical data. Simple Bonferroni correction for multiple hypothesis testing was applied for adjustment at a two-sided alpha level of 0.0042 (=0.05/12).

Survival analyses were performed using Kaplan–Meier survival estimates with the log-rank test. Cox proportional hazards regression analysis was performed to analyze the associations of survival with other factors. The model included the following variables: sex (male vs. female), age, tumor size, primary tumor location (right-sided colon vs. left-sided colon vs. rectum), pT stage (pT2 vs. pT3 vs. pT4), tumor histology (well to moderately differentiated vs. poorly differentiated), mucin production (positive vs. negative), lymph node metastasis (positive vs. negative), peritoneal metastasis (positive vs. negative), distant organ metastasis (positive vs. negative), surgical status (complete vs. incomplete resection), and CD70/POSNT expression status (double-positive vs. others). Backward elimination with a threshold of *p* = 0.05 was used to select variables for the final model.

### 4.5. Gene Mutation Analyses

*BRAF* V600E mutation analyses were performed by polymerase chain reaction–direct sequencing. Sequences of primers used were as follows: *BRAF* forward, tgc ttg ctc tga tag gaa aat g; *BRAF* reverse, cag ggc caa aaa ttt aat cag t. *KRAS* mutation status was collected from the medical records.

### 4.6. Cells, Plasmids, and Reagents

Human colon fibroblast CCD-18Co and HCT-116 cells were purchased from the American Type Culture Collection (ATCC, Manassas, VA, USA). Cells were maintained in Dulbecco’s modified Eagle’s medium supplemented with 10% fetal bovine serum (FBS).

The lentiviral vectors for full-length human POSTN, human CD70, and control LacZ expression with blasticidin or hygromycin B resistance genes in CCD-18Co cells were constructed using the CSII-CMV-MCS-IRES2-Bsd plasmid, which was kindly provided by Dr. H. Miyoshi (RIKEN BioResource Center, Tsukuba, Japan).

### 4.7. Cellular Proliferation and Co-Culture Migration and Invasion Assays

CCD-18Co cells with or without CD70 and POSTN and their control counterparts expressing LacZ were seeded into 12-well plates (5 × 10^3^). After incubation, cell numbers were measured using CellTiter 96^®^ Aqueous One Solution (Promega, Madison, WI, USA) according to the manufacturer’s protocol. 

Co-culture migration assays were performed using the Falcon^®^ Permeable Support for 24-well plates with 8.0 µm transparent PET membrane (Corning, NY, USA) according to the manufacturer’s procedure. First, 1 × 10^4^ of CCD-18Co cells with LacZ, CD70, and/or POSTN were seeded into 24-well plates. After 24 h incubation, 2 × 10^4^ enhanced-green fluorescent protein (EGFP)-labeled HCT-116 cells were added to the migration chamber. After additional incubation for 24 h, the migrated EGFP-positive HCT-116 cells on the opposite side of the PET membranes were counted under a fluorescent microscope. Ten-percent FBS was used as a chemoattractant.

Co-culture invasion assays were performed using the Corning^®^ BioCoat™ Matrigel^®^ invasion chambers with 8.0 µm PET membrane (Corning, Corning, NY, USA) according to the manufacturer’s procedure. First, 1 × 10^4^ of CCD-18Co cells with or without CD70 and POSTN were incubated in an invasion chamber. After 24 h incubation, 4 × 10^4^ of EGFP-labeled HCT-116 cells were added to the upper chamber. After additional incubation for 48 h, the invaded EGFP-positive HCT-116 cells were counted under a fluorescent microscope. Ten-percent FBS was used as a chemoattractant.

### 4.8. Immunoblot Analyses

Immunoblot analyses were performed as previously described [15,48,49]. In brief, whole-cell lysates were prepared using 1× sodium dodecyl sulfate (SDS) sample buffer containing 50 mM Tris-HCl and 2% SDS. For POSTN, cultured medium was used for analyses. Proteins separated by SDS polyacrylamide gel electrophoresis were transferred to a PVDF membrane. Antibody dilutions are summarized in Supplementary Table S2. Signal intensity was measured by ImageJ software (NIH).

## Figures and Tables

**Table 1:** Characteristics of CRC with or without CD70 and POSTN expression in CAFs.

	Total No.		Characteristics of CRC CAFs								*p*-Value	
			CD70+/POSTN+		CD70+/POSTN−		CD70−/POSTN+		CD70−/POSTN−			
	269	(100%)	21	(8%)	19	(7%)	98	(36%)	131	(49%)		
Sex											0.88	a
Male	143	(53%)	10	(48%)	9	(47%)	52	(53%)	72	(55%)		
Female	126	(47%)	11	(52%)	10	(53%)	46	(47%)	59	(45%)		
Age, years (mean ± S.D.)	59.2 ± 11.9		64.8 ± 16.6		67.6 ± 11.3		70.4 ± 11.4		68.0 ± 12.9		0.23	b
Size, cm (mean ± S.D.)	5.0 ± 2.6		5.3 ± 2.7		5.0 ± 2.7		5.2 ± 2.4		4.8 ± 2.7		0.63	b
Tumor location											0.33	a
Right-sided colon	124	(46%)	12	(57%)	8	(42%)	44	(45%)	60	(46%)		
Left-sided colon	86	(32%)	3	(14%)	9	(47%)	29	(30%)	45	(34%)		
Rectum	59	(22%)	6	(29%)	2	(11%)	25	(26%)	26	(20%)		
pT stage											0.032	a
T2	36	(13%)	0	(0%)	1	(5%)	8	(8%)	27	(21%)		
T3	189	(70%)	18	(86%)	15	(79%)	70	(71%)	86	(66%)		
T4	44	(16%)	3	(14%)	3	(16%)	20	(20%)	18	(14%)		
Histological differentiation											0.45	a
Well to moderately	242	(90%)	19	(90%)	19	(100%)	86	(88%)	118	(90%)		
Poorly	27	(10%)	2	(10%)	0	(0%)	12	(12%)	13	(10%)		
Mucus production											0.26	a
Positive	14	(5%)	0	(0%)	0	(0%)	8	(8%)	6	(5%)		
Negative	255	(95%)	21	(100%)	19	(100%)	90	(92%)	125	(95%)		
Lymph node metastasis											0.30	a
Positive	124	(49%)	13	(68%)	7	(39%)	46	(49%)	58	(48%)		
Negative	129	(51%)	6	(32%)	11	(61%)	48	(51%)	64	(52%)		
Peritoneal metastasis											0.0059	a
Positive	50	(19%)	7	(33%)	0	(0%)	25	(26%)	18	(14%)		
Negative	219	(81%)	14	(67%)	19	(100%)	73	(74%)	113	(86%)		
Distant organ metastasis											0.0020	a
Positive	50	(19%)	7	(33%)	0	(0%)	23	(23%)	14	(11%)		
Negative	219	(81%)	14	(67%)	19	(100%)	75	(77%)	117	(89%)		
Operation status											0.0011 ^†^	a
Complete resection	237	(88%)	13	(62%)	18	(95%)	86	(88%)	120	(92%)		
Incomplete resection	32	(12%)	8	(38%)	1	(5%)	12	(12%)	11	(8%)		
MMR system status											0.25	a
Deficient	31	(12%)	1	(5%)	0	(0%)	12	(12%)	18	(14%)		
Proficient	238	(88%)	20	(95%)	19	(100%)	86	(88%)	113	(86%)		

^a^ Chi-squared or ^b^ one-way ANOVA testing was used to calculate *p*-values. The Bonferroni-corrected *p*-value for significance was *p* = 0.0042 (0.05/12). ^†^, post hoc tests revealed significant differences between CD70+/POSTN+ and CD70−/POSTN− (*p* = 0.0032).

**Table 2:** Characteristics of immune cells in the CRC microenvironment classified by CD70 and POSTN expression in CAFs.

	Total No.		Characteristics of CAFs								*p*-Value
			CD70+/POSTN+		CD70+/POSTN−		CD70−/POSTN+		CD70−/POSTN−		
	269	(100%)	21	(8%)	19	(7%)	98	(36%)	131	(49%)	
CD4+ TILs (/HPF)	11 (4–24)		12 (9–18)		10 (3.5–27)		13 (6–26.5)		8 (4–19.5)		0.15
CD8+ TILs (/HPF)	12 (4–24)		8 (3–13)		10 (3.5–17)		9 (3–21)		15 (5.5–33)		0.0063
CD27+ TILs (/HPF)	32 (12–82)		21 (5–45)		35 (12.5–83)		27 (7.25–61.5)		36 (17.5–103.5)		0.21
PD-1+ TILs (/HPF)	28.5 (15.0–52.3)		27.0 (14.0–47.0)		17.0 (14.5–35.5)		27.0 (13.3–50.0)		32.5 (19.0–56.3)		0.35
FOXP3+ TILs (/HPF)	38 (21–66)		35 (21–54)		39 (21–65)		34 (16–54)		42 (26–74)		0.13
CD68+ TAMs (pixels)	1794 (1076–3002)		2843 (2411–3356)		2138 (1711–3567)		2037 (1191.5–3836.75)		1493 (869–2399)		<0.0001
CD163+ TAMs (pixels)	944 (372–2043)		1126 (833–2395)		940 (389–1842.5)		981 (312–2568.75)		875 (352.5–1732)		0.23

Kruskal–Wallis testing was used for the analyses. Data are shown as median (25th–75th percentiles). The Bonferroni-corrected *p*-value for significance was *p* = 0.0071 (0.05/7). TILs, tumor-infiltrating lymphocytes; TAMs, tumor-associated macrophages; HPF, high-power field (×400).

**Table 3:** Multivariable Cox hazards analysis of colorectal cancer patients.

	Hazard Ratio	95% CI		*p*-Value
		Min	Max	
Well to moderately differentiated histology	0.25	0.13	0.47	<0.0001
Age (<70)	0.50	0.29	0.86	0.012
Lymph node metastasis	1.98	1.14	3.45	0.015
CD70+/POSTN+ in CAFs	3.78	1.83	7.83	0.00035
Peritoneal metastasis	5.45	3.05	9.73	<0.0001

The multivariable Cox hazards analysis model initially included sex, age, primary tumor location, tumor size, pT stage, surgery status, tumor histology, mucus production, lymph node metastasis, distant organ metastasis, peritoneal metastasis, mismatch repair system status, and immunohistochemistry for CD70 and POSTN (double positive vs. others). A backward elimination with a threshold of *p* = 0.05 was used to select variables in the final model.

## Data Availability

The datasets used and/or analyzed during the present study are available from the corresponding author on reasonable request.

## Supplementary Materials

The following supporting information can be downloaded at: https://www.mdpi.com/article/10.3390/ijms25052537/s1.
