# Supplementary material for: Simultaneous Expression of CD70 and POSTN in Cancer-Associated Fibroblasts Predicts Worse Survival of Colorectal Cancer Patients
Source: Int J Mol Sci. 2024 Feb 22;25(5):2537. doi: 10.3390/ijms25052537 (PMC10931655; doi:10.3390/ijms25052537)
Supplement: Supplementary file 1 [file ijms-25-02537-s001.zip › ijms-2829949-supplementary.pdf]

International Journal of *Molecular Sciences*

MDPI

Simultaneous Expression of CD70 and POSTN in Cancer-Associated Fibroblasts Predicts Worse Survival of Colorectal Cancer Patients

Masayuki Komura, Chengbo Wang, Sunao Ito, Shunsuke Kato, Akane Ueki, Masahide Ebi, Naotaka Ogasawara, Toyonori Tsuzuki, Kenji Kasai, Kunio Kasugai, Shuji Takiguchi, Satoru Takahashi and Shingo Inaguma

Supplementary Table S1. Gene mutation and p53 expression classified by CD70 and POSTN expressions in CAFs.

	Characteristics of CRC											
	Total No.		CD70+/POSTN+		CD70+/POSTN−		CD70−/POSTN+		CD70−/POSTN-		P-value	
	262	[100%]	20	[8%]	18	[3%]	97	[50%]	127	[39%]		
KRAS/*BRAF* status											0.98	a
Wild type	15	(39%)	1	(33%)	1	(100%)	7	(37%)	6	(40%)		
KRAS mutants	19	(50%)	2	(67%)	0	(0%)	10	(53%)	7	(47%)		
BRAF mutants	4	(11%)	0	(0%)	0	(0%)	2	(11%)	2	(13%)		
p53 expression											0.073	b
Wild-type pattern	59	(23%)	1	(5%)	4	(22%)	17	(18%)	37	(29%)		
Over expression	143	(55%)	11	(55%)	11	(61%)	54	(56%)	67	(53%)		
Cytoplasmic expression	10	(4%)	0	(0%)	0	(0%)	4	(4%)	6	(5%)		
Complete loss	50	(19%)	8	(40%)	3	(17%)	22	(23%)	17	(13%)		

P-value was calculated by [a]Fisher's exact or [b]chi-square test.

Supplementary Table S2. Antibodies and conditions for immunohistochemistry and immunoblotting.

		IHC		IB	
Genes	Reagents	Retrieval methods	Dilution	Dilution	Antibodies
ACTA2	IF	pH 9.0, 20 min	2,000	1,000	EPR5368, Abcam (Cambridge, UK)
ACTA2	IF	pH 9.0, 20 min	600	-	Clone 1A4, Dako/Agilent, (Santa Clara, CA)
ACTB	-	-	-	5,000	Clone AC-74, Merck KGaA (Darmstadt, Germany)
CCNA	IV	pH 8.5, 64 min	100	-	sc-751, Santa Cruz Biothechnology, Inc. (Dallas, TX)
CD27	BP	pH 9.0, 20 min	80	-	Clone 137B4, Novocastra/Leica biosystems (Nussloch, Germany)
CD4	BP	pH 9.0, 20 min	100	-	4B12, Leica Biosystems (Wetzlar, Germany)
CD8	BP	pH 9.0, 20 min	200	-	C8/144B, NICHIREI BIOSCIENCES INC. (Tokyo, Japan)
CD68	BP	pH 9.0, 20 min	2 (Pre-diluted)	-	Clone PG-M1, NICHIREI BIOSCIENCES INC. (Tokyo, Japan)
CD70	BP, IF	pH 9.0, 20 min	400	-	Clone #301731, R&D systems (Minneapolis, MN)
CD70	-	-	-	1,000	#72094, Cell Signaling Technology, Inc. (Danvers, MA)
CD163	BP	pH 6.0, 20 min	200	-	10D6, Leica Biosystems (Bannockburn, IL)
FAP	IF	pH 9.0, 20 min	200	1,000	EPR20021, Abcam (Cambridge, UK)
FOXP3	BP	pH 9.0, 20 min	250	-	Clone 236A/E7, Abcam (Cambridge, UK)
GMNN	OV	pH 8.5, 64 min	500	-	EPR14637, Abcam (Cambridge, UK)
Ki-67	OV	pH 8.5, 64 min	100	-	Clone MIB-1, Dako/Agilent, (Santa Clara, CA)
MLH1	OV	pH 8.5, 64 min	200	-	Clone G168-728, BD Biosciences, (Franklin Lakes, NJ)
MMP2	-	-	-	1,000	Clone D4M2N, Cell Signaling Technology, Inc. (Danvers, MA)
MSH2	OV	pH 8.5, 64 min	200	-	Clone G219-1129, BD Biosciences, (Franklin Lakes, NJ)
MSH6	OV	pH 8.5, 64 min	400	-	Clone 44/MSH6, BD Biosciences, (Franklin Lakes, NJ)
p53	BP	pH 9.0, 20 min	500	-	Clone DO7, Leica Biosystems (Wetzlar, Germany)
PD-1	OV	pH 8.5, 64 min	200	-	Clone EPR4877(2), Abcam (Cambridge, UK)
PDPN	IF	pH 9.0, 20 min	10,000	1,000	EPR22182, Abcam (Cambridge, UK)
POSTN	BP, IF	pH 9.0, 20 min	100	1,000	EPR92460, Abcam (Cambridge, UK)
PHH3	BP	pH 9.0, 20 min	500	-	Cell Marque™, Millipore SIGMA, (Rocklin, CA)
PMS2	OV+Linker	pH 9.0, 20 min	50	-	Clone A16-4, BD Biosciences, (Franklin Lakes, NJ)
P-STAT3 (Tyr705)	-	-	-	1,000	Clone D3A7, Cell Signaling Technology, Inc. (Danvers, MA)
P-STAT3 (Ser727)	-	-	-	1,000	#9134S, Cell Signaling Technology, Inc. (Danvers, MA)
STAT3	-	-	-	1,000	#8768, Cell Signaling Technology, Inc. (Danvers, MA)

BP, Leica BOND Polymer Refine Detection; IV, Ventana iView reagent; OV, Ventana OptiView reagent. IF, immunofluorescent.

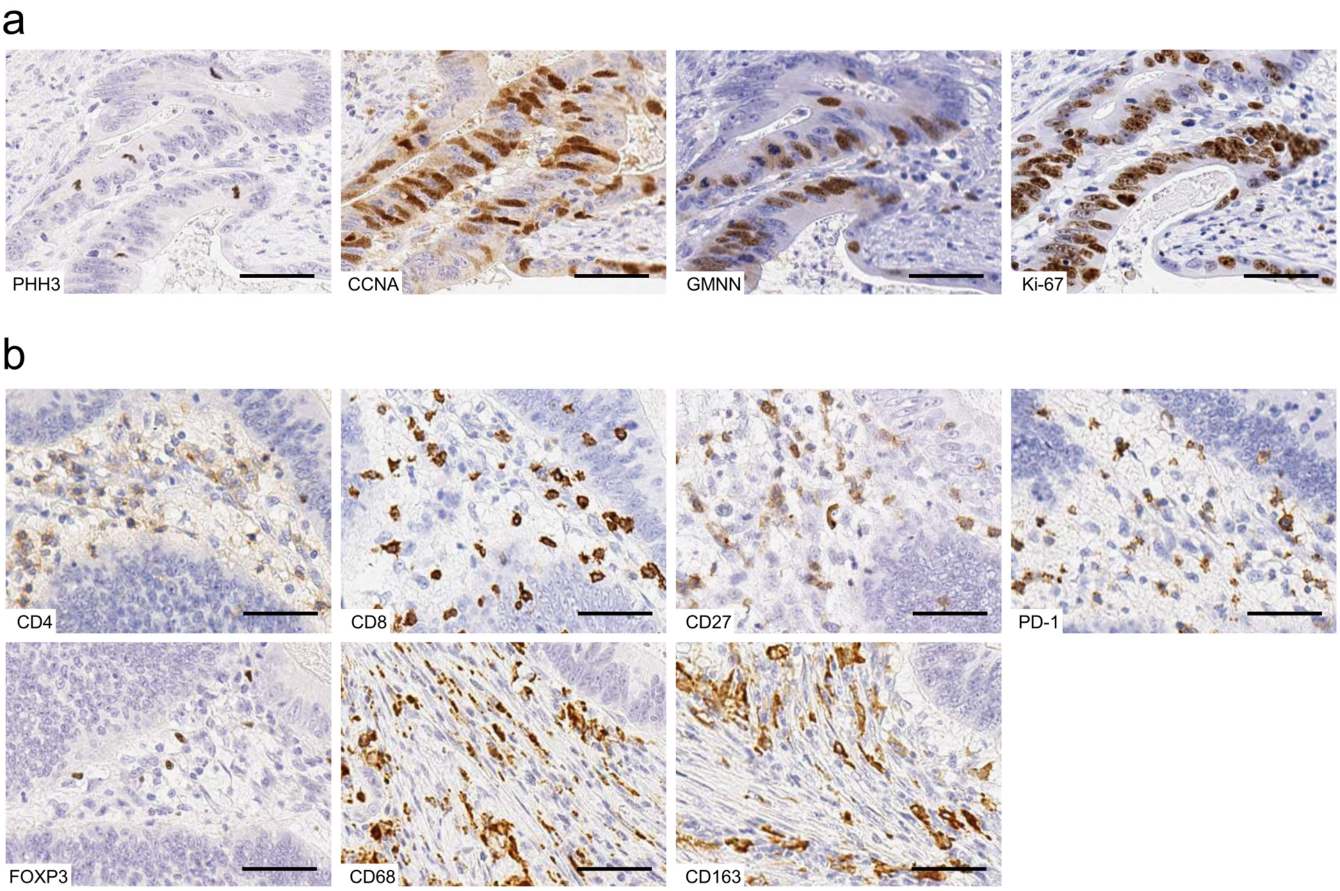

Supplementary Figure S1. Representative images for immunohistochemistry. a, Images for cellular proliferation markers. b, Images for immune cell markers. Bar, 50 μm
